# A Spatial, Social and Environmental Study of Tuberculosis in China Using Statistical and GIS Technology

**DOI:** 10.3390/ijerph120201425

**Published:** 2015-01-27

**Authors:** Wenyi Sun, Jianhua Gong, Jieping Zhou, Yanlin Zhao, Junxiang Tan, Abdoul Nasser Ibrahim, Yang Zhou

**Affiliations:** 1 State Key Laboratory of Remote Sensing Science, Institute of Remote Sensing and Digital Earth, Chinese Academy of Sciences, Beijing 100101, China; E-Mails: ygxddz@qq.com (W.S.); gongjh@radi.ac.cn (J.G.); nasen@radi.ac.cn (A.N.I.); 2 University of Chinese Academy of Sciences, Beijing 100049, China; 3 Zhejiang-CAS Application Center for Geoinformatics, Zhejiang 314100, China; 4 The Chinese Center for Disease Control and Prevention, Beijing 102206, China; E-Mails: zhaoyanlin@chinatb.org (Y.L.Z.); zhouyang@chinatb.org (Y.Z.); 5 Center for Airborne Remote Sensing, Institute of Remote Sensing and Digital Earth, Chinese Academy of Sciences, Beijing 100094, China; E-Mail: tanjx@radi.ac.cn

**Keywords:** tuberculosis, partial least squares path model, geographically weighted regression

## Abstract

Tuberculosis (TB) remains a major public health problem in China, and its incidence shows certain regional disparities. Systematic investigations of the social and environmental factors influencing TB are necessary for the prevention and control of the disease. Data on cases were obtained from the Chinese Center for Disease and Prevention. Social and environmental variables were tabulated to investigate the latent factor structure of the data using exploratory factor analysis (EFA). Partial least square path modeling (PLS-PM) was used to analyze the complex causal relationship and hysteresis effects between the factors and TB prevalence. A geographically weighted regression (GWR) model was used to explore the local association between factors and TB prevalence. EFA and PLS-PM indicated significant associations between TB prevalence and its latent factors. Altitude, longitude, climate, and education burden played an important role; primary industry employment, population density, air quality, and economic level had hysteresis with different lag time; health service and unemployment played a limited role but had limited hysteresis. Additionally, the GWR model showed that each latent factor had different effects on TB prevalence in different areas. It is necessary to formulate regional measures and strategies for TB control and prevention in China according to the local regional effects of specific factors.

## 1. Introduction

As a major cause of human illness and death, tuberculosis (TB) remains one of the world’s principal infectious diseases. In 2007, the World Health Organization (WHO) observed more than 9 million new TB cases globally (more than 50% of new cases occurred in Asia), and approximately 1.76 million people died from this disease [1]. China is one of the 22 TB high-burden countries. According to WHO TB annual reports, with 12% of all TB cases in the world, China ranked second only to India [2]. In the past 20 years, China has successfully reduced the morbidity and mortality of TB, with the prevalence of smear-positive TB decreasing from 134/100,000 individuals in 1990 to 66/100,000 individuals in 2010 and mortality decreasing from 19.1/100,000 individuals in 1990 to 3.9/100,000 individuals in 2010 [3]. However, TB has always been ranked among the top five on the national list of notifiable infectious diseases.

Various factors affect the prevalence of TB. Previous studies have found that the prevalence of TB is associated with individual differences, such as genetic susceptibility [4], sex [5], education [6], race [7,8], migration [9], drinking alcohol [10], smoking [11], and related diseases [12,13,14]. Additionally, at the ecological level, geographic, climatic and socio-economic factors also impact TB prevalence. These factors include elevation [15], climate [16], air pollution [11], the national economic level [17], unemployment rate [10], poverty [18], and social instability [19]. Compared to individual studies [4,5,6,7,8,9,10,11,12,13,14], ecological studies on TB prevalence are relatively insufficient [10,11,15,16,17,18,19], especially in China. These ecological studies primarily investigated the effects of certain factors on TB prevalence and did not systematically address the comprehensive effects of these factors. Consequently, it is necessary to carry out systematic ecological research to determine a comprehensive relationship between ecological factors and TB prevalence in China, including geographic, climatic and socio-economic factors, to provide substantial amounts of information to support TB control and prevention. 

Since 1979, China has conducted five TB epidemiology surveys. These surveys were conducted in 1979, 1985, 1990, 2000, and 2010. In the latest survey, the epidemiology of TB in China showed a geographically unbalanced pattern. The results indicated that the prevalence in the eastern region was the lowest (65/100,000 individuals). Moreover, the prevalence in the western region was the highest (198/100,000 individuals), more than three times the rate in the eastern region [3]. Furthermore, ecological factors, such as climate, geography, and socio-economic factors, commonly show spatial autocorrelation and heterogeneity [20], which indicate that the influence would also show spatial heterogeneity. Many previous studies have used traditional multivariate regression models to determine the global relationships between TB prevalence and selected factors. These regression approaches have included linear regression [18], logistic regression [7], and negative binomial regression [9]. However, it is difficult to incorporate spatial heterogeneity analysis of the effects into these traditional models. Therefore, investigating the role of spatial heterogeneity in the relationship between risk factors and TB prevalence is essential for developing TB control and prevention policies. 

Consequently, based on the previous studies, the present study explored geographic, climatic, and socio-economic factors and employed methods of statistical and spatial analysis to evaluate the role of spatial heterogeneity in the complex ecological causes of TB prevalence, for the purpose of providing essential information for TB control and prevention.

## 2. Materials and methods 

### 2.1. Data Sources 

**Figure 1:**
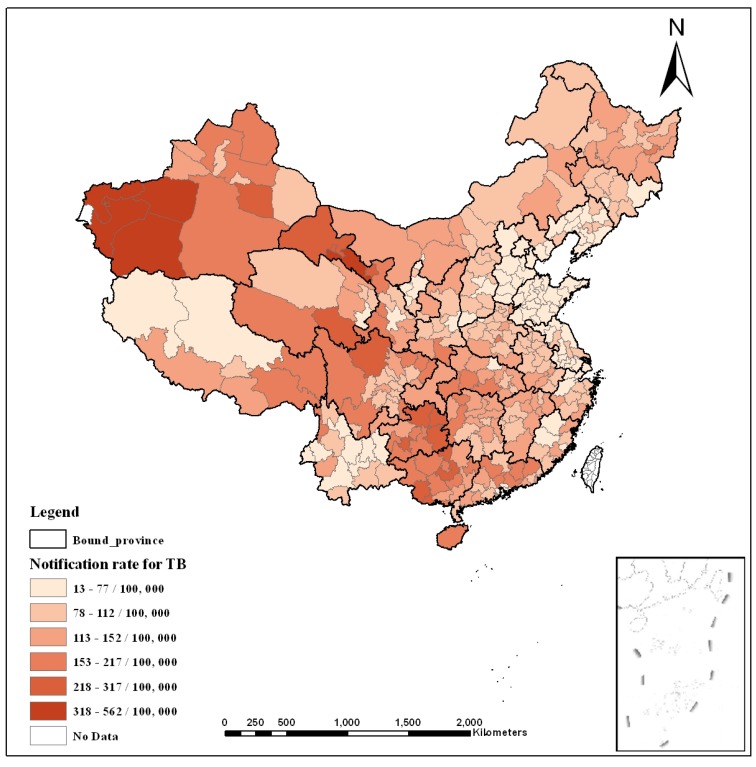
Average annual notification rate (per 100,000 population) for TB in China in 2007.

To strengthen national TB prevention and control, the Disease Control Bureau of the Ministry of Health and the Chinese Center for Disease Control and Prevention launched a TB management information system in 2005 [21]. Patient details are reported directly to the system over the network, and statistics are added to the public health science database. The TB prevalence data in this study were obtained from that system for 1 January 2007 to 31 December 2007. The climatic and geographic data (including e.g., elevation, temperature, and precipitation) were collected from the website for the China Meteorological Data Sharing Service System. Air quality data (including the air pollution index (API)) were obtained from the Ministry of Environmental Protection of China. Socio-economic data were gathered from provincial government websites. To investigate the hysteresis of the effects, we also obtained these variables from 2002 to 2006. The spatial unit for this study is the municipality, which is one of China’s administrative divisions; a total of 337 municipalities existed in 2007. Table 1 describes the observed variables and their data sources. Figure 1 shows the annual notification rate of TB in 2007. 

**Table 1:** Specification of the observed variables and latent risk factors.

Observed Variable	Description of Observed Variable	Data Source	Period	Latent Risk Factor	% of Variance
X4	Annual average precipitation (mm)	Meteorological Data Sharing Service System of China	2002–2007	Climatic factor	93.2%
X7	Annual average temperature (°C)		2002–2007		
X8	Annual average vapor pressure (Pa)		2002–2007		
X9	Annual average relative humidity (%)		2002–2007		
X10	Annual average minimum temperature (°C)		2002–2007		
X11	Annual average maximum temperature (°C)		2002–2007		
X12	Number of days in per year in which precipitation is greater than 0.1 mm (day)		2002–2007	Rainy day factor	100%
X5	Average altitude (m)		2002–2007	Altitude factor	98.7%
X1	Annual average air pressure (Pa)		2002–2007		
X3	Average longitude (degrees)		2002–2007	Longitude factor	100%
X15	Air pollution index (API)	Ministry of Environmental Protection of China	2002–2007	Air quality	100%
X16	Per capita annual net income of rural residents (RMB yuan)	China Regional Economic Statistical Yearbook	2002–2007	Economic level	88.2%
X17	Per capita annual cost-of-living expense of rural residents (RMB yuan)		2002–2007		
X18	Per capita annual disposable income of urban residents (RMB yuan)		2002–2007		
X19	Per capita annual cost-of-living expense of urban residents (RMB yuan)		2002–2007		
X20	Per capita annual gross domestic product (RMB yuan)		2002–2007		
X22	Per capita annual fixed time deposit of urban and rural residents (RMB yuan)		2002–2007		
X26	Annual unemployment rate of urban residents (%)		2002–2007	Unemployment level	100%
X27	Number of students per teacher of primary school		2002–2007	Education burden	89.4%
X28	Number of students per teacher of ordinary high school		2002–2007		
X30	Population density (population/km^2^)		2002–2007	Population density	100%
X23	Percentage of primary industry employees from the total number of employees (%)		2002–2007	Primary industry employment	93.6%
X36	Percentage of primary industry employees from the total number of employees in rural areas (%)		2002–2007		
X34	Number of beds in medical institutions per thousand people		2002–2007	Health service	97.3%
X35	Number of medical workers per thousand people		2002–2007		

### 2.2. Statistical Methods 

To explore the latent structure of the above variables, we used exploratory factor analysis (EFA) [22] to extract the latent synthetic risk factors using SPSS V.21.0 (SPSS Inc., Chicago, IL, USA). The objective of EFA is to determine a small number of common factors to explain the joint variability of a set of input variables [22]. In view of the possible occurrence of non-normality and multi-collinearity, and based on the EFA result, we chose the partial least squares path model (PLS-PM) [23] to construct a structure equation model (SEM) to analyze the complex relationships between latent risk factors and TB prevalence. PLS path modeling was designed by Wold [24,25] to analyze high-dimensional data in a low-structure environment. PLS techniques have undergone various extensions and modifications. This method has been used successfully for other diseases or health problems, such as coronary heart disease [26] and multidrug-resistant tuberculosis (MDR-TB) [27]. Because it can accommodate a variety of sampling distributions and small sample sizes, PLS path modeling is known as a “flexible model” [28]. SmartPLS v2.0 was created to build PLS path models as part of a project at the Institute of Operations Management and Organizations (School of Business), University of Hamburg (Germany). In this study, the path-weighting scheme was applied to obtain the inner estimates of the standardized latent variables in the PLS-PM, and the resampling number for bootstrapping was set at 1000. The standardized scores of the latent risk factors were then estimated for further analysis. In this study, to examine the hysteresis of the factors, we also analyzed the complex relationships between factors and TB prevalence using variables from the five previous years.

### 2.3. Analysis Using a Geographical Statistical Model 

Based on the latent variable scores from the PLS-PM, the geographically weighted regression (GWR) model was implemented to explore the local spatial heterogeneity of the causal relationships between TB prevalence and latent risk factors in the year of the largest effect. The GWR model is a local spatial regression model that generates parameters resolved by the spatial units of analysis. In this model, the regression coefficients show the local spatial variation, and the standard errors of the coefficients illustrate the reliability of the estimated coefficients [29]. This process allows an evaluation of the spatial heterogeneity in the estimated associations between the independent and dependent variables. GWR v4.0 [30] was used to apply a GWR model with various combinations of fixed/adaptive bandwidth and Gaussian/bi-square kernel to choose the most suitable model. Considering the samples are not regularly spaced in our study area, we implemented the adaptive bandwidth and Gaussian kernel to build the model, which provides the same number of samples for each local estimate and is based on the whitepaper for GWR [31,32]. We also selected the golden-section search option to automatically search for the best bandwidth size. To show the results of the GWR model, maps were created using ArcGIS v10.2 [33] with the Albers projection option.

## 3. Results and Discussion

### 3.1. Extraction of Latent Risk Factors 

The latent risk factors, including “Climatic factor”, “Altitude factor”, “Longitude factor”, “Air quality”, “Rainy day factor”, “Education burden”, “Primary industry employment”, “Population density”, “Economic level”, “Unemployment level” and “Health service”, were extracted from the observed variables (Table 1) by EFA. “Climatic factor” was based on annual average precipitation (X4), annual average temperature (X7), annual average vapor pressure (X8), annual average relative humidity (X9), annual average minimum temperature (X10) and annual average maximum temperature (X11) and could explain approximately 93.2% of the total variance of these variables. “Altitude factor” was based on average altitude (X1) and annual average air pressure (X5) and explained 98.7% of the variance. “Longitude factor” was simply based on by average longitude (X3). “Air quality” was solely based on the annual average air pollution index (X15). “Rainy day factor” was also solely based on the number of rainy days with precipitation greater than 0.1 mm (X12). “Education burden” was based on the number of students per teacher of primary school (X27) and number of students per teacher of ordinary high school (X28) and explained approximately 89.4% of the total variance. “Primary industry employment” was based on the percentage of primary industry employees from the total number of employees (X23) and the percentage of primary industry employees from the total number of employees in rural areas (X36) and explained 93.6% of the variance. “Population density” was solely based on population density (X30). “Economic level” was determined by the per capita annual net income of rural residents (X16), per capita annual cost of living expense of rural residents (X17), per capita annual disposable income of urban residents (X18), per capita annual cost of living expense of urban residents (X19), per capita annual gross domestic product (X20) and per capita annual fixed time deposit of urban and rural residents (X22) and explained 88.2% of the variance. “Unemployment level” was based solely on the annual unemployment rate of urban residents (X26). “Health service” was determined by the number of beds in medical institutions per thousand people (X34) and number of medical workers per thousand people (X35) and explained 97.3% of the variance. Furthermore, the dependent factor, named “TB prevalence”, was solely based on TB incidence.

### 3.2. Complex Relationship between TB Prevalence and Latent Risk Factors 

**Figure 2:**
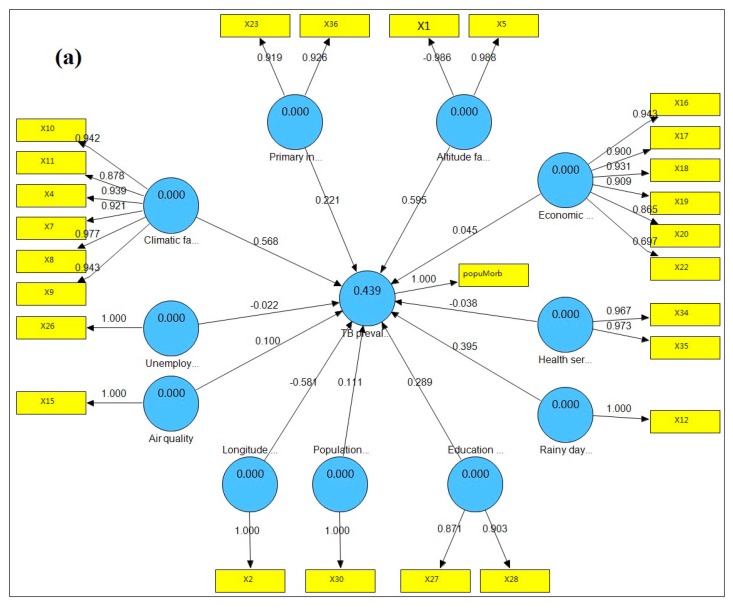

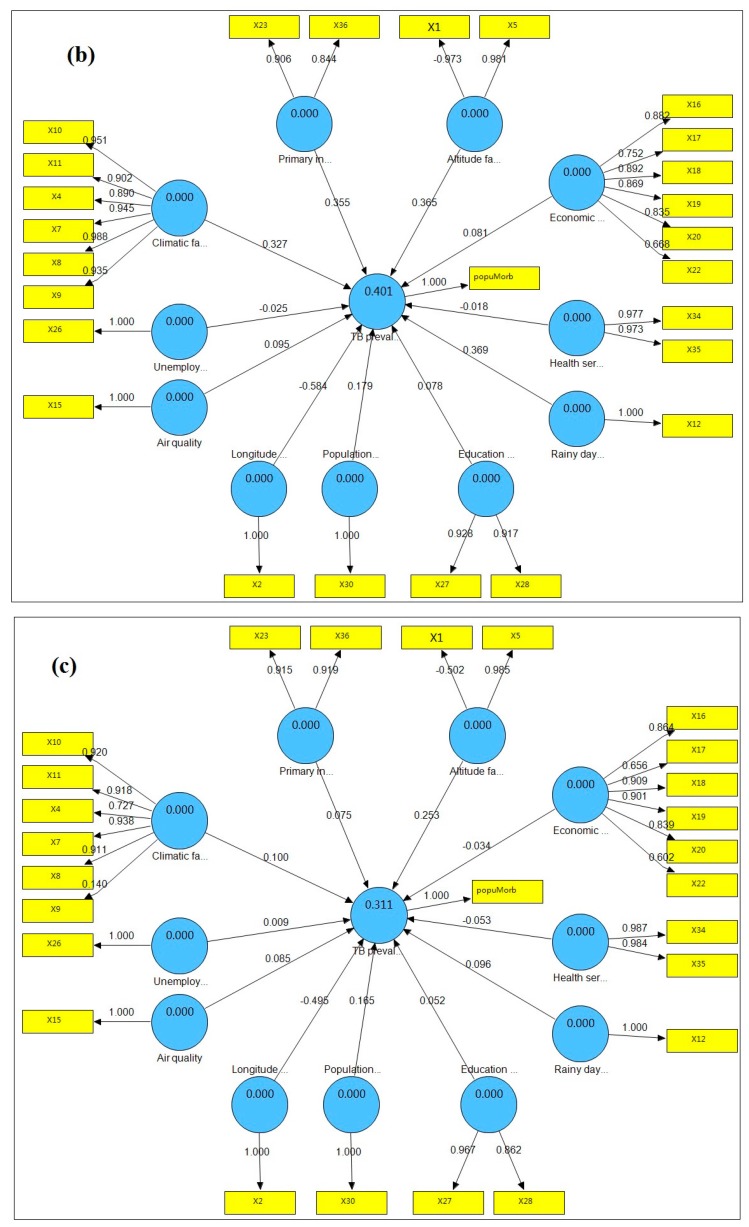

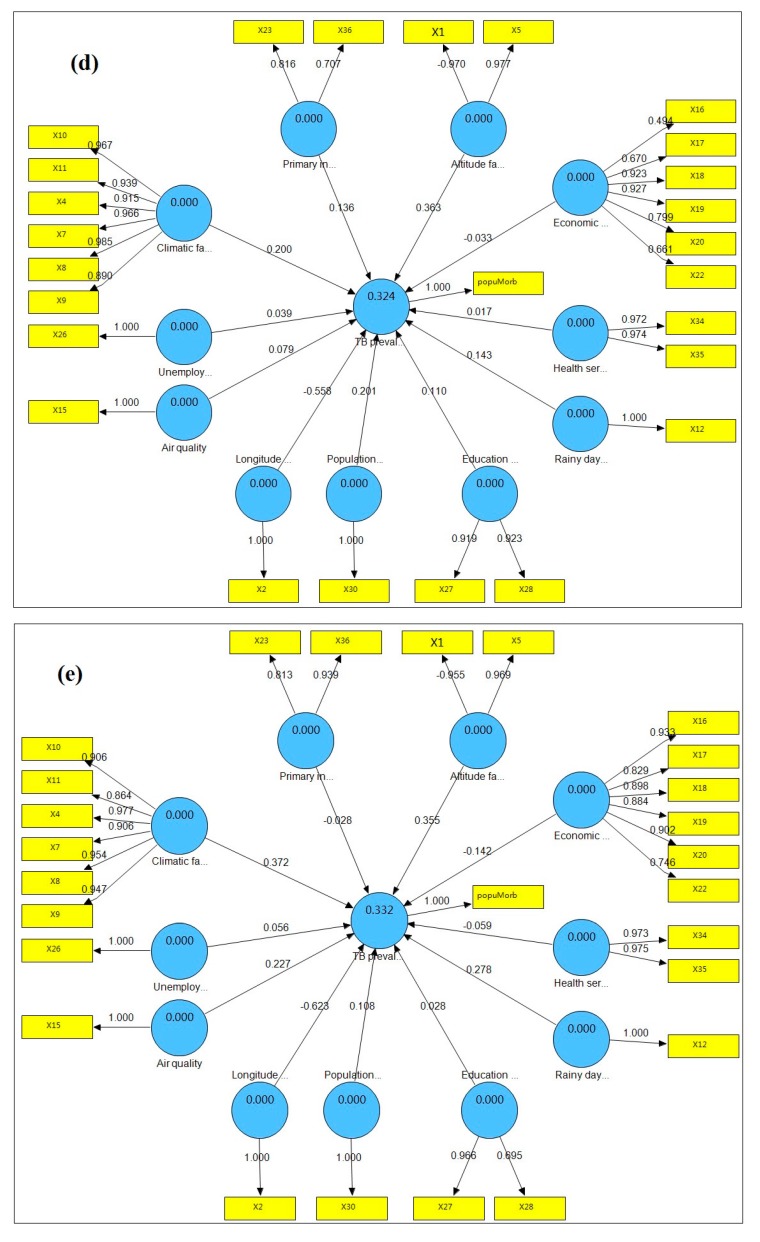

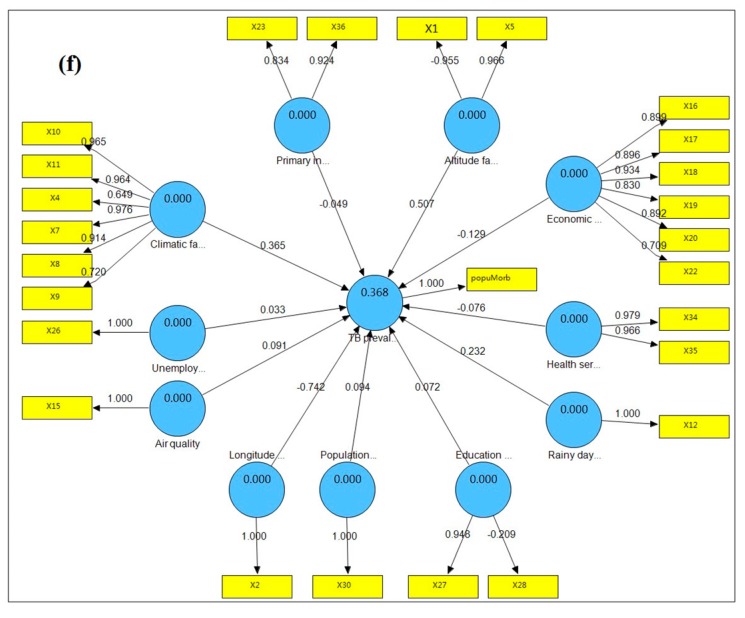
PLS path models of TB prevalence with its latent risk factors. (**a**) TB prevalence (2007) with factors (2007); (**b**) TB prevalence (2007) with factors (2006); (**c**) TB prevalence (2007) with factors (2005); (**d**) TB prevalence (2007) with factors (2004); (**e**) TB prevalence (2007) with factors (2003); (**f**) TB prevalence (2007) with factors (2002).

The PLS path model of TB prevalence and its latent risk factors is shown in Figure 2a. The model shows the contribution of the latent risk factors to TB prevalence as well as the contribution of the variables to the latent risk factors. The R^2^ of the model is 0.439, showing that these latent risk factors could explain 43.9% of the total variance of “TB prevalence”. Of these factors, “Altitude factor” had the largest significant effect on “TB prevalence”, with a standardized path coefficient of 0.595, indicating a positive relationship between “TB prevalence” and “Altitude factor”. “Longitude factor” had the second-largest influence on “TB prevalence”, with a standardized path coefficient of -0.581, indicating a negative relationship between “TB prevalence” and “Longitude factor”. “Climatic factor”, “Rainy day factor”, “Education burden”, “Primary industry employment”, “Population density”, “Air quality”, and “Economic level” all had positive influences on “TB prevalence”, with standardized path coefficients of 0.568, 0.395, 0.289, 0.221, 0.111, 0.100, and 0.045, respectively. “Health service” and “Unemployment level” had negative influences on “TB prevalence”, with coefficients of −0.038 and −0.022, respectively.

The bootstrapping test results for the outer weights and outer loadings of variables are shown in supplementary files (Tables S1 and S2), and the bootstrapping test results for path coefficients of the latent risk factors are shown in Table 2. All of the outer loadings of the variables were significant at a 0.001 level (*p* < 0.001), and most outer weights for the variables were significant at a 0.01 level (*p* < 0.01), indicating that most of the observed variables reflected their latent risk factor at an adequate level. “Climatic factor”, “Education burden”, “Altitude factor”, “Longitude factor” and “Rainy day factor” were significant at a 0.01 level (*p* < 0.01); “Population density” and “Primary industry employment” were significant at a 0.05 level (*p* < 0.05). These results demonstrated that these seven latent risk factors had major influences and played an important role in TB prevalence, whereas “Air quality”, “Economic level”, “Unemployment level” and “Health service” had limited effects.

**Table 2:** Bootstrapping tests of path coefficients of latent risk factors from the PLS-PM.

Structural Model	Original Sample	Sample Mean	Standard Deviation	Standard Error	T Statistics
Air quality → TB prevalence	0.1002	0.0757	0.0587	0.0587	1.4915
Climatic factor → TB prevalence	0.5681	0.5353	0.225	0.225	2.8004 **
Education burden → TB prevalence	0.2887	0.2454	0.0664	0.0664	3.5616 ***
Primary industry employment → TB prevalence	0.2208	0.1814	0.1007	0.1007	1.9476 *
Altitude factor → TB prevalence	0.5953	0.5947	0.1558	0.1558	4.1515 ***
Health service → TB prevalence	−0.0380	−0.0151	0.08	0.08	0.0047
Population density → TB prevalence	0.1109	0.1344	0.0595	0.0595	1.9689 *
Longitude factor → TB prevalence	−0.5811	−0.5112	0.1031	0.1031	5.0916 ***
Rainy day factor → TB prevalence	0.3946	0.3982	0.151	0.151	3.0139 **
Economic level → TB prevalence	0.0452	0.035	0.0931	0.0931	0.404
Unemployment → TB prevalence	−0.0221	−0.009	0.0545	0.0545	0.2817

*** *p* < 0.005, ** *p* < 0.01, * *p* < 0.05.

### 3.3. Hysteresis of the Relationship between TB Prevalence and Latent Risk Factors 

To determine the hysteresis of these complex relationships, the variables from the five previous years were also included. Figure 2b–f show the PLS path models of TB prevalence with its latent risk factors from the five previous years and the standardized path coefficients illustrating the hysteresis of the factors from the five previous years. The R^2^ of the PLS path model for 2007 was the largest (0.439), indicating that the latent risk factors from 2007 explained 43.9% of the total variance for “TB prevalence” and had the largest contribution. We can determine the hysteresis of the latent risk factors based on the variation of the standardized path coefficients between latent risk factors and TB prevalence from 2007 to 2002. The standardized path coefficients of “Altitude factor”, “Climatic factor”, “Rainy day factor” and “Education burden” for 2007 were the largest, showing that there was limited hysteresis between these factors and “TB prevalence”. The standardized path coefficient of “Primary industry employment” for 2006 was the largest, showing that there was a 1-year lag time between “Primary industry employment” and “TB prevalence”. The standardized path coefficient of “Population density” for 2004 was the largest, showing that there was a 3-year lag time between “Population density” and “TB prevalence”. The standardized path coefficients of “Air quality” and “Economic level” for 2003 were the largest, showing that there was a 4-year lag time between these factors and “TB prevalence”. The standardized path coefficients of “Unemployment” and “Health service” were always small and not significant from 2007 to 2002, showing that these factors played a limited role and had limited hysteresis. 

### 3.4. Local Spatial Heterogeneity of the Relationship 

Because the R^2^ of the PLS path model for 2007 was the largest, we selected the latent risk factors for 2007 to include in the GWR model. Table 3 summarizes the statistical outcomes of the GWR model between TB prevalence and its latent risk factors in 2007. In the GWR model, R^2^ was 0.526, indicating that the model explained 52.6% of the variance in TB prevalence. According to the Akaike information criterion with a correction (AICc), a model selection criterion based on Fotheringham [34], the GWR AICc value (775.28) was lower than the ordinary least squares (OLS) AICc value (800.86), and the difference between the two AICc values was greater than 3. This result showed that the performance of the GWR model was better than that of the OLS model. Furthermore, an analysis of variance (ANOVA) also showed that the GWR model was better than the OLS model, as the model fit at a significant level (F = 5.34, *p* < 0.05). A bandwidth size of 82 was selected by the golden-section search, and this value was appropriate for the model, meaning that there were 82 samples provided for each local estimation within the adaptive Gaussian kernel. Moran’s I for residuals was 0.033 and was not significant at a 0.05 level (Z-score = 1.676, *p* = 0.0937), indicating that the residuals were spatially random.

**Table 3:** Parameter estimates for the GWR model.

Parameter	Min	1st Quartile	Median	3rd Quartile	Max	Mean
Intercept	−0.1539	−0.1130	−0.0686	−0.0364	−0.0126	−0.0751
Air quality	−0.1400	−0.0534	−0.0041	0.0375	0.0994	−0.0108
Climatic factor	0.0686	0.1466	0.1976	0.2443	0.2877	0.1896
Economic level	−0.1156	−0.0655	−0.0461	−0.0179	0.0250	−0.0462
Education burden	−0.0239	−0.0074	0.0099	0.0244	0.0444	0.0088
Health service	0.0217	0.0718	0.1264	0.1699	0.2015	0.1201
Altitude factor	−0.0366	−0.0180	−0.0079	0.0158	0.0432	−0.0020
Unemployment level	−0.6484	−0.5595	−0.5170	−0.4698	−0.2393	−0.4965
Longitude factor	−0.2530	−0.1798	−0.0865	−0.0312	0.0175	−0.1039
Primary industry employment	0.0084	0.0623	0.0978	0.1426	0.1769	0.0979
Rainy day factor	0.1669	0.3046	0.3496	0.4271	0.5821	0.3633
Population density	−0.0285	−0.0124	0.0073	0.0281	0.0521	0.0089

R^2 ^= 0.526, adjusted R^2 ^= 0.461, AICc = 775.28.

**Figure 3:**
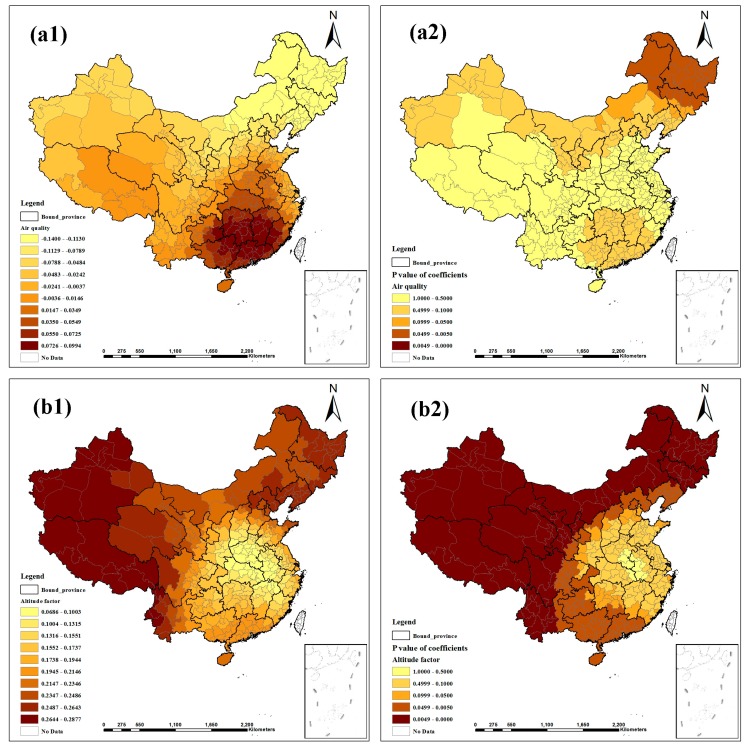

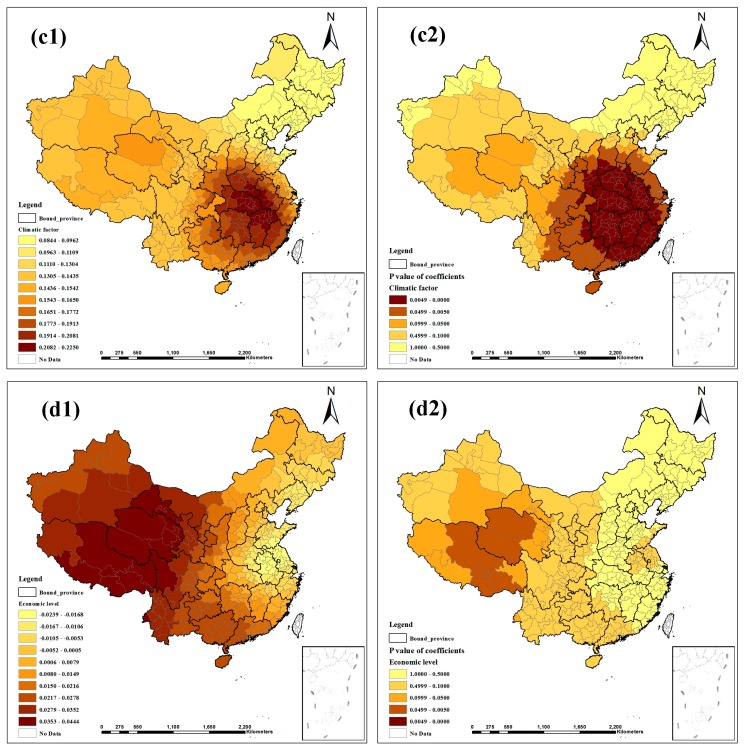
Spatial heterogeneity of the factor coefficients for TB prevalence derived from the GWR model. ((**a1**–**d1**) distribution of coefficients of “Air quality”, “Altitude factor”, “Climatic factor”, and “Economic level”. (**a2**–**d2**) distribution of *p* values of coefficients).

**Figure 4:**
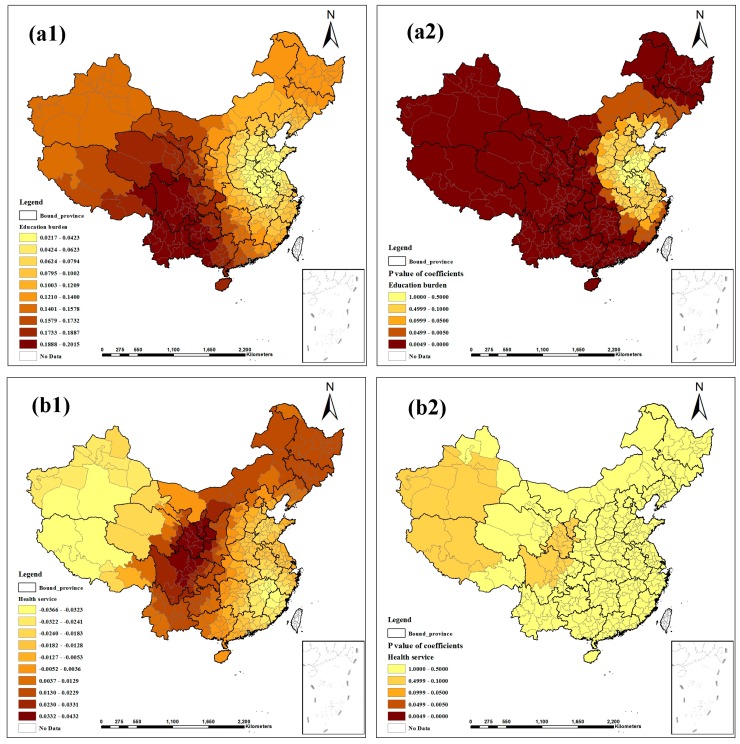

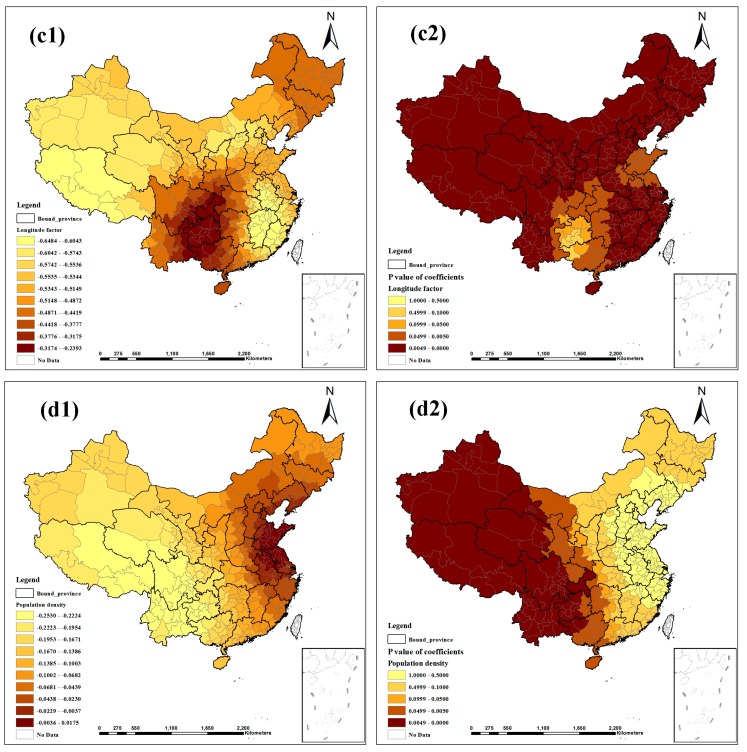
Spatial heterogeneity of the factor coefficients for TB prevalence derived from the GWR model ((**a1**–**d1**) distribution of coefficients of “Education burden”, “Health service”, “Longitude factor”, and “Population density”. (**a2**–**d2**) distribution of *p* values of coefficients).

**Figure 5:**
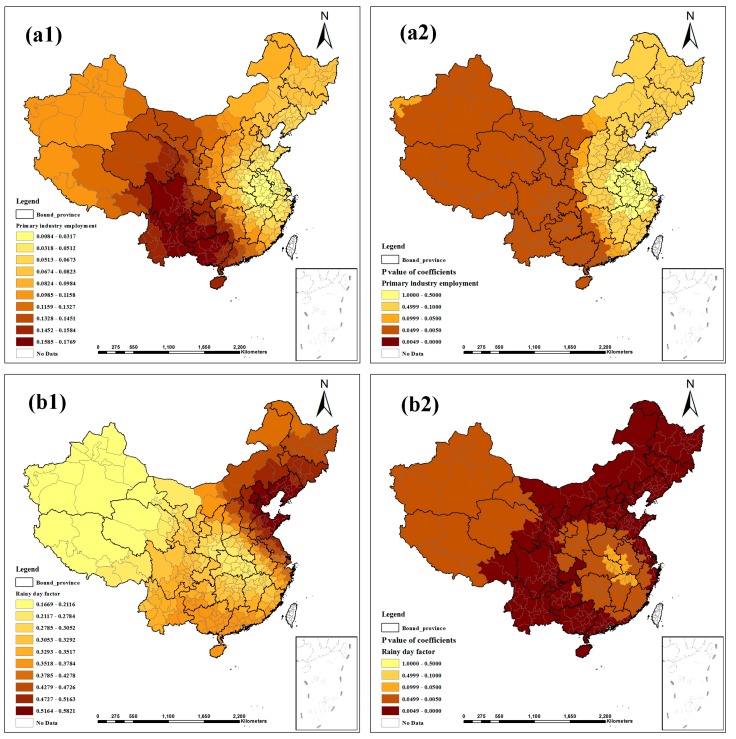

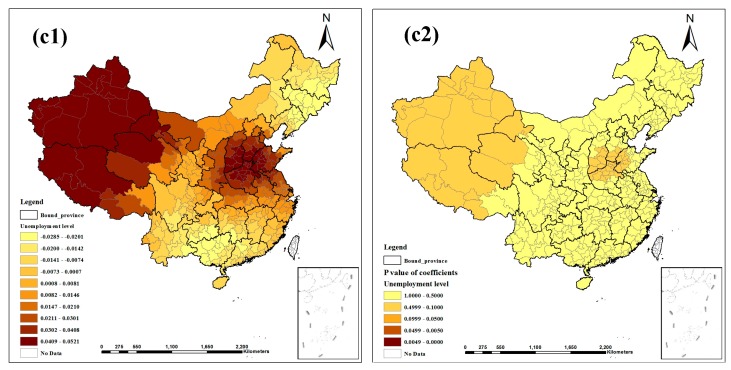
Spatial heterogeneity of the factor coefficients for TB prevalence derived from the GWR model. ((**a1**–**c1**) distribution of coefficients of “Primary industry employment”, “Rainy day factor”, and “Unemployment level”. (**a2**–**c2**) distribution of *p* values of coefficients).

Figure 3, Figure 4 and Figure 5 show the contour maps for the regression coefficients of the latent risk factors and their *p* values. These results clearly demonstrate the existence of a local unstable spatial dependence between TB prevalence and its latent risk factors. In Central China and South China, the standardized regression coefficient estimates of “Air quality” were positive, and the rest were negative; only the regression coefficients in Northeast China were significant (Figure 3a1, a2). “Altitude factor” had positive effects on “TB prevalence” and had larger effects in the northern regions; the regression coefficients were primarily significant, except in East China and Central China (Figure 3b1, b2). The standardized regression coefficient estimates of “Climatic factor” were all positive; however, only the regression coefficients in East China, Central China and South China were significant (Figure 3c1,c2). In Northeast China, East China and North China, the standardized regression coefficient estimates of “Economic level” were negative, and the rest were positive; most estimates were not significant (Figure 3d1, d2). “Education burden” had positive effects on “TB prevalence” and had larger effects in the western regions; the regression coefficients were primarily significant except in East China and North China (Figure 4a1, a2). The standardized regression coefficient estimates of “Health service” were primarily negative, except in Northwest China, North China and Northeast China, but the estimates were not significant (Figure 4b1, b2). “Longitude factor” had negative effects on “TB prevalence”, and had larger effects in Northwest China and East China; most estimates were significant (Figure 4c1, c2). The standardized regression coefficient estimates of “Population density” were negative in most regions; only the regression coefficients in Northwest China, Southwest China and South China were significant (Figure 4d1, d2). “Primary industry employment” had positive effects on “TB prevalence” and had larger effects in the southern regions, but only the regression coefficients in Northwest China, Southwest China and South China were significant (Figure 5a1, a2). The standardized regression coefficient estimates of “Rainy day factor” were mostly positive, and most estimates were significant (Figure 5b1, b2). The associations between “Unemployment level” and “TB prevalence” were mostly negative except in Northwest China and North China; most estimates were not significant (Figure 5c1, c2).

## 4. Discussion

Previously, many methods have been used to study the relationships between TB prevalence and the factors that influence it. These methods include correlation coefficient analysis [35], generalized linear mixed models [36], log-linear regression models [37] and negative binomial regression models [9]. However, these methods do not consider the latent relationship between the variables, which can be addressed by the PLS-PM. In this study, we adopted the PLS-PM to analyze the complex relationship between TB prevalence and its latent factors. The PLS-PM is referred to as a soft-modeling technique with minimal demands in terms of measurement scale, sample size and residual distribution [24], and it can make full use of data to explain the inherent characteristics of the observed variables [23]. To examine the hysteresis of these complex relationships, the variables for the five previous years were also used in the analysis. 

We found that the environmental factors substantially impacted “TB prevalence”. First, two geographic factors (“Altitude factor” and “Longitude factor”) significantly affected “TB prevalence”, indicating that serious TB prevalence arose in regions with higher altitude, lower air pressure and lower longitude. However, several contrasting findings were observed and indicated that TB incidence decreased with increasing altitude [38,39]. Mansoer *et al.*, who found this negative association, have also stated that this association was not explained by potential confounders as indicators of socio-economic status [39]. There are special circumstances in China. The areas located at higher longitudes are in the plains, near the sea, at a relatively low altitude, and they have developed economies and medical facilities. Therefore, there was less TB incidence in these regions. The areas located at lower longitudes are in hilly or mountainous inland areas, at a relatively higher altitude, with underdeveloped economies and a shortage of medical facilities. Therefore, the TB incidence was higher in these regions. This difference may explain the positive association between “Altitude factor” and “TB prevalence” and the reason that our results were inconsistent with those of other studies in this case [38,39]. Second, “Climatic factor” and “Rainy day factor” also had complex impacts on “TB prevalence”. More hot and humid weather was estimated to increase TB prevalence (*i.e*., higher temperature, more precipitation, more rainy days, higher vapor pressure and higher relative humidity). The bacillus *Mycobacterium tuberculosis* reproduces more readily in hot and humid weather. In addition, due to the high humidity, air circulation is poor, furnishing conditions for the spread of TB. Guidi *et al.* also found that hot weather and humidity increased TB prevalence in the summer and autumn in Ferrara (Italy) [40]. Finally, although no strong relationship was observed between “Air quality” and “TB prevalence” in 2007, a 4-year lag time of positive effect was observed. As “Air quality” was only determined by the annual average air pollution index, the positive effect indicated that poor air quality had a positive effect on TB prevalence. A team of researchers from the University of Medicine and Dentistry of New Jersey (USA) found that exposure to polluted air may cause the human body’s cells to react slowly or to be non-reactive, thus weakening their ability to resist the threats posed by *Mycobacterium tuberculosis* [41]. Another study found evidence that passive smoking and indoor air pollution increased the risk of TB [11].

We also found that social factors as well as environmental factors were relevant to TB prevalence. First, “Education burden” had a positive effect on “TB prevalence”, indicating that it was associated with increased TB prevalence when there were more students per teacher in primary school and ordinary high school. School areas are crowded areas, and adolescence is the period during which the incidence of TB is high. Thus, one TB patient can spread the disease. Second, “Primary industry employment” positively affected “TB prevalence”, with a 1-year lag time. An increasing ratio of primary industry employees to total employees was associated with increased TB prevalence. These results are similar to those of the 5^th^ national TB epidemiological survey of China [3], which reported that 71.3% of patients were rural patients and that 83% of these rural patients were engaged in farming, forestry, animal husbandry and fishery (primary industries) or were agricultural laborers. People engaged in primary industries always have low incomes, limited medical knowledge and poor living conditions; all of these would accelerate the spread of TB. Third, although “Economic level” and “Population density” had limited effects on “TB prevalence”, hysteresis effects on “TB prevalence” were observed over years. Serious TB prevalence arose in the regions with lower economic levels and lower population density. Several previous studies have reported similar findings [42,43,44]. Finally, we did not find a significant relationship or hysteresis between “Unemployment level” and “TB prevalence”, contrary to the results of many other studies [10,43,44,45]. The public unemployment rate in China is mainly derived from the unemployment rate of urban residents; in comparisons with other countries, the unemployment rate of rural residents was not included in the calculations. This difference may be the primary explanation for this result.

These ecological factors played a role in “TB prevalence”. The results of the GWR model illustrated that the impacts of these factors differed among the studied regions. The contour maps of the results from the GWR model visually demonstrate the complex, spatially dependent relationships between TB prevalence and its latent risk factors. These relationships could explain the causes of regional variation in TB prevalence in China. A few points deserve special attention. First, in Southwest China, Northwest China and Northeast China, “Altitude factor” had a greater positive effect on “TB prevalence”, a result similar to those of the 5th national TB epidemiological survey of China [3]. The report stated that serious TB prevalence primarily developed in the Western region of the country, where the altitudes are relatively high. Due to the unique topography of Southwest China, Northwest China and Northeast China, the population inhabits mountainous regions in which natural conditions make living difficult and limit transportation. As local economic and social development is not balanced, many areas in these regions do not administer the Bacille Calmette Guerin (BCG) vaccination. This difference may explain the greater positive correlation found in these regions [46]. Second, in East China, South China and Central China, “Climatic factor” had a greater positive effect on “TB prevalence”. These regions have a tropical or subtropical monsoon climate, with hot, humid weather and poor air circulation, which may be conducive to the spread of TB. Third, “Population density” had a greater negative association with “TB prevalence” in Southwest China and Northwest China. These regions are undeveloped over wide areas and sparsely populated, with a scattered population and limited transportation. The medical resources in these regions cannot meet the local health needs [46]. These characteristics may be the cause of the greater negative correlation found in these regions. Finally, “Primary industry employment” had a greater positive association with “TB prevalence” in Southwest China and Northwest China. As the regional economy in these regions is not developed, most local residents are engaged in primary industries, with little income and no medical security. Many TB patients cannot afford the cost of treatment and always delay treatment. This situation is conducive to the spread of TB [3]. These spatially dependent relationships illustrate that regional planning and strategies must be formulated for TB control and prevention based on the spatial variation of the factors.

This study has several limitations. First, the data used were collected from a variety of sources, such as the national weather database and the regional economic statistical yearbook. The methods used to process these data may have differed, biasing the results. Second, TB data collected over one year were used to analyze the influence of spatial factors. However, the epidemiology focuses on not only the influence of space but also the influence of time. Therefore, in the future, we will also focus on the influence of temporal or spatiotemporal factors to obtain a deeper understanding. Third, due to the difficulty of collecting data, the observed variables may not completely reflect the latent factors. The “Air quality” latent factor should also be based on the concentrations of nitrogen dioxide, inhalable particulates and sulfur dioxide, but data on these variables have only been released in China since September 2008. Similarly, since more cases occurred in rural areas, the unemployment rate for rural residents should be included in the “Unemployment level” latent factor, but data on this rate have not been published. Because of these limitations, more studies that investigate larger or different regions are needed to add to and compare with the findings of this study.

## 5. Conclusions 

We found that climate, altitude, longitude, primary industry employment, population density, education burden and economic level impacted TB prevalence to varying degrees. Moreover, each factor had different effects on TB prevalence in different regions in China. Therefore, regional measures and strategies for control and prevention should be established according to the local spatially dependent relationships between TB prevalence and these factors.

## Supplementary Files

Supplementary File 1

## List of Abbreviations

TB: tuberculosis
WHO: World Health Organization
API: air pollution index
EFA: exploratory factor analysis
PLS-PM: partial least squares path model
SEM: structure equation model
MDR-TB: multidrug-resistant tuberculosis
GWR: geographically weighted regression
AICc: Akaike information criterion with a correction
OLS: ordinary least squares
ANOVA: analysis of variance
BCG: Bacille Calmette Guerin
