# Supplementary material for: A Spatial, Social and Environmental Study of Tuberculosis in China Using Statistical and GIS Technology
Source: Int J Environ Res Public Health. 2015 Jan 27;12(2):1425–48. doi: 10.3390/ijerph120201425 (PMC4344675; doi:10.3390/ijerph120201425)
Supplement: Supplementary File 1 [file ijerph-12-01425-s001.pdf]

Supplementary Information

A Spatial, Social and Environmental Study of Tuberculosis in China Using Statistical and GIS Technology

Table S1. Bootstrapping tests of outer loadings of observed variables from the PLS-PM.

Observed Variable	Sample Mean	Standard Deviation	Standard Error	T Statistics
X1 <− Altitude factor	−0.9863	0.0057	0.0057	172.9124 *
X5 <− Altitude factor	0.9885	0.0044	0.0044	222.7768 *
X2 <− Longitude factor	1	-	-	-
X4 <− Climatic factor	0.8817	0.1531	0.1531	6.1318 *
X7 <− Climatic factor	0.8763	0.157	0.157	5.8669 *
X8 <− Climatic factor	0.9221	0.1509	0.1509	6.4738 *
X9 <− Climatic factor	0.8749	0.2048	0.2048	4.6056 *
X10 <− Climatic factor	0.8937	0.1526	0.1526	6.1708 *
X11 <− Climatic factor	0.8385	0.1655	0.1655	5.3061 *
X12 <− Rainy day factor	1	-	-	-
X15 <− Air quality	1	-	-	-
X16 <− Economic level	0.9428	0.0065	0.0065	144.9075 *
X17 <− Economic level	0.8975	0.0189	0.0189	47.7 *
X18 <− Economic level	0.9305	0.0092	0.0092	100.8193 *
X19 <− Economic level	0.9092	0.0126	0.0126	72.3518 *
X20 <− Economic level	0.866	0.0305	0.0305	28.3325 *
X22 <− Economic level	0.7098	0.0368	0.0368	18.9368 *
X23 <− Primary industry employment	0.9185	0.0172	0.0172	53.4403 *
X36 <− Primary industry employment	0.9254	0.0143	0.0143	64.9397 *
X26 <− Unemployment level	1	-	-	-
X27 <− Education burden	0.8704	0.0301	0.0301	28.8949 *
X28 <− Education burden	0.9002	0.0363	0.0363	24.835 *
X30 <− Population density	1	-	-	-
X34 <− Health service	0.9651	0.0358	0.0358	26.9949 *
X35 <− Health service	0.9722	0.0318	0.0318	30.5485 *

* $p < 0.001$.

Table S2. Bootstrapping tests of outer weights of observed variables from the PLS-PM.

Observed Variable	Sample Mean	Standard Deviation	Standard Error	T Statistics
X1 <− Altitude factor	−0.4819	0.0279	0.0279	17.3917 ***
X5 <− Altitude factor	0.5307	0.0263	0.0263	20.0668 ***
X2 <− Longitude factor	1.0000	-	-	-
X4 <− Climatic factor	0.1471	0.1901	0.1901	0.8548
X7 <− Climatic factor	0.1205	0.1106	0.1106	1.0302
X8 <− Climatic factor	0.1967	0.0768	0.0768	2.7435 **
X9 <− Climatic factor	0.3390	0.4316	0.4316	0.9409
X10 <− Climatic factor	0.1484	0.0764	0.0764	1.9656 *
X11 <− Climatic factor	0.0380	0.2705	0.2705	0.0566
X12 <− Rainy day factor	1.0000	-	-	-
X15 <− Air quality	1.0000	-	-	-
X16 <− Economic level	0.2413	0.0142	0.0142	17.1478 ***
X17 <− Economic level	0.1850	0.0153	0.0153	12.2181 ***
X18 <− Economic level	0.2127	0.0165	0.0165	12.9425 ***
X19 <− Economic level	0.1489	0.0181	0.0181	8.2803 ***
X20 <− Economic level	0.1776	0.0190	0.0190	9.3876 ***
X22 <− Economic level	0.1670	0.0236	0.0236	6.8784 ***
X23 <− Primary industry employment	0.5324	0.0347	0.0347	15.2770 ***
X36 <− Primary industry employment	0.5511	0.0360	0.0360	15.3755 ***
X26 <− Unemployment level	1.0000	0.0000	0.0000	0.0000
X27 <− Education burden	0.5268	0.0609	0.0609	8.6324 ***
X28 <− Education burden	0.5999	0.0574	0.0574	10.4487 ***
X30 <− Population density	1.0000	-	-	-
X34 <− Health service	0.4834	0.0989	0.0989	4.9875 ***
X35 <− Health service	0.5449	0.0914	0.0914	5.8780 ***

*** $p < 0.001$, ** $p < 0.01$, * $p < 0.05$.

© 2015 by the authors; licensee MDPI, Basel, Switzerland. This article is an open access article distributed under the terms and conditions of the Creative Commons Attribution license (http://creativecommons.org/licenses/by/4.0/).
